# Improved linear classifier model with Nyström

**DOI:** 10.1371/journal.pone.0206798

**Published:** 2018-11-05

**Authors:** Changming Zhu, Xiang Ji, Chao Chen, Rigui Zhou, Lai Wei, Xiafen Zhang

**Affiliations:** College of Information Engineering, Shanghai Maritime University, Shanghai, China; Institut de Robotica i Informatica Industrial, SPAIN

## Abstract

Most data sets consist of interlaced-distributed samples from multiple classes and since these samples always cannot be classified correctly by a linear hyperplane, so we name them nonlinearly separable data sets and corresponding classifiers are named nonlinear classifiers. Traditional nonlinear classifiers adopt kernel functions to generate kernel matrices and then get optimal classifier parameters with the solution of these matrices. But computing and storing kernel matrices brings high computational and space complexities. Since INMKMHKS adopts Nyström approximation technique and NysCK changes nonlinearly separable data to linearly ones so as to reduce the complexities, we combines ideas of them to develop an improved NysCK (INysCK). Moreover, we extend INysCK into multi-view applications and propose multi-view INysCK (MINysCK). Related experiments validate the effectiveness of them in terms of accuracy, convergence, Rademacher complexity, etc.

## Introduction

### Background

In real-world applications, most data sets consist of interlaced-distributed samples from multiple classes. If samples cannot (can) be classified correctly with a linear hyperplane, we name them nonlinearly (linearly) separable samples. As we know, linear classifiers including HK, MHKS, and SVM [1] are feasible to process linearly separable samples. While for nonlinearly ones which are ubiquitous, nonlinear classifiers including NCC [2], FC-NTD [3], KMHKS [4], KSVM [5] are more suitable. One kind of nonlinear classifiers is kernel-based ones including MultiV-KMHKS [6], MVMHKS [7], RMVMHKS [8], DLMMLM [9], UDLMMLM [10], etc [11–13] and they adopt kernel functions to generate kernel matrices firstly and get optimal classifier parameters after the solution of these matrices. Here, for convenience, we summary full names and abbreviations for some terms in Table 1.

**Table 1:** Full name and abbreviation for some used terms.

Full name	Abbreviation
Ho-Kashyap algorithm	HK
Ho-Kashyap algorithm with squared approximation of the misclassification errors	MHKS
support vector machine	SVM
nonlinearly combined classifiers	NCC
fuzzy clustering with nonlinearly transformed data	FC-NTD
kernelized modification of MHKS	KMHKS
kernel SVM	KSVM
multi-views KMHKS	MultiV-KMHKS
multi-view learning developed from single-view patterns with Ho-Kashyap linear classification strategy	MVMHKS
regularized MVMHKS	RMVMHKS
double-fold localized multiple matrix learning machine	DLMMLM
Universum based DLMMLM	UDLMMLM
Nyström approximation matrix with multiple KMHKSs	NMKMHKS
improved NMKMHKS	INMKMHKS
cluster kernel	CK
Nyström CK	NysCK
improved NysCK	INysCK
multi-view INysCK	MINysCK
multi-view L2-SVM	MSVM
multiple graph regularized generative model	MGGM
multi-view least squares support vector machines	MV-LSSVM
multi-view and multi-feature learning	MVMFL
semi-supervised multi-view maximum entropy discrimination approach	SMVMED
multi-view low-rank sparse subspace clustering	MLRSSC
kernel MLRSSC	KMLRSSC
multi-view kernel spectral clustering	MVKSC
matrix-pattern-oriented MHKS with boundary projection discrimination	BPDMatMHKS
regularized weighted least square support vector classifier	rWLSSVC
novel dissimilarity learning	NDL
locality constrained dictionary learning	LCDL
scale-invariant feature transform	SIFT
singular value decomposition	SVD
radial basis function	RBF

### Problem and previous solutions

Most kernel-based classifiers cost an *O*(*n*^3^) computational complexity to decompose matrices and an *O*(*Mn*^2^) space complexity to store them where *n* is number of samples and *M* is number of used kernel functions. But the complexities are too high for most real-world classification problems. Fortunately, some classifiers including NMKMHKS [14], INMKMHKS [11], and NysCK which is developed on the base of cluster kernel (CK) [15] are developed to reduce complexities. (1) NMKMHKS selects *s* samples from *n* ones and uses Nyström approximation technique to get approximation form for each kernel matrix. With NMKMHKS, computational complexity can be reduced to *O*(*Mns*^2^) and space complexity can be reduced to *O*(*n*^2^). While since the numbers and parameters of used kernel functions should be initialized beforehand and *s* is set in random, the performance of NMKMHKS maybe poor when comes to noise cases and is sensitive to *s*. (2) INMKMHKS adopts clustering technology to guide the generations of kernel functions and approximation matrices. This operation can solve the defects of NMKMHKS and keep a lower complexity. (3) NysCK decomposes each kernel matrix ***K*** by ***K*** = ***FF***^*T*^ where each row in ***F*** represents a linearly separable sample and then nonlinearly separable samples can be changed to linearly ones. [15] has validated that those linearly ones correspond to the original ones and they can be classified by linear classifiers with a high accuracy.

### Motivation and novelty

Since INMKMHKS avoids the setting of *s* and kernel parameters and NysCK changes the nonlinearly separable samples to linearly ones, we combine them in together to develop improved NysCK (INysCK) to reduce complexities further. Moreover, multi-view data set which consists of samples with multiple views and each view consists of multiple features is a widely used one in real world and many corresponding multi-view classifiers are developed [16–18]. Since INysCK has not an ability to process multi-view data sets, thus we extend INysCK into multi-view applications and propose multi-view INysCK (MINysCK).

Since INMKMHKS (NysCK) was developed in 2015 (2017), thus ideas and innovations of them are still new at some extends. What’s more, to the best of our knowledge, until now, there is no method combines their ideas in together. In other words, the idea of our methods is novel and it is the first trial for this. In our methods, for the original data set, we first adopt the ideas of INMKMHKS to generate several kernel functions and get the corresponding Nyström approximation matrices. Then on the base of these matrices, we adopt the ideas of NysCK to get ***F***. In ***F***, each row represents a linearly separable sample which corresponds to an original sample. Then we can classify these linearly separable samples with linear classifiers. This operation is similar with the one of classifying the original samples with some nonlinear classifiers. Moreover, this operation won’t influence the classification results.

### Contribution

Contributions of our work are (1) provide a new idea to process nonlinear classification problems and needn’t to initialize many parameters beforehand; (2) keep low computational and space complexities; (3) first time to process multi-view problems with such an idea.

## Related work

### Nyström approximation technique

For a kernel-based classifier, whether the solution is feasible or not depends on the eigendecomposition of kernel matrix and in general, the eigendecomposition needs a *O*(*n*^3^) computational cost where *n* is number of samples. In order to cut down the computational cost, [19] develops Nyström approximation technique to speed up the eigendecomposition. Simply speaking, one selects *s* samples from the whole data set to approximate the kernel matrix, then computational complexity can be reduced to *O*(*ns*^2^). Recently, Nyström approximation technique has been applied into multiple different fields. For example, [20] uses this technique to get approximate infinite-dimensional region covariance descriptor which significantly outperforms the low-dimensional descriptors on image classification task; [21] introduces Nyström into kernel subspace learning and reduces the time and space complexities; [22] combines Nyström method with spectral clustering algorithm to decrease the computation complexity of the spectral clustering with a high clustering accuracy kept.

### NysCK

Suppose there is a nonlinearly separable data set ***X*** = [***X***_***l***_, ***X***_***v***_], ***X***_***l***_ consists of *l* labeled samples, ***X***_***v***_ consists of *v* unlabeled samples, *l* < *v*, *n* = *l* + *v*. Objective of NysCK is changing nonlinearly separable samples to linearly ones with Nyström approximation technique and predicting class labels of those unlabeled ones. For these *n* samples, NysCK constructs a kernel matrix ***K*** firstly and then selects *s* samples from ***X***_*l*_ to decompose ***K*** with $K=[\begin{array}{cc} W & K_{12}\\ K_{21} & K_{22} \end{array}]$ and $C=(\begin{array}{c} W\\ K_{21} \end{array})$ where $W\in R^{s\times s}$ corresponds to the *s* samples. After that, NysCK carries out SVD on ***W*** and gets the Nyström approximation matrix of ***K***, i.e., $\tilde{K}=CW_{k}^{†}C^{T}\approx K$ where $W_{k}^{†}$ denotes the pseudo-inverse of ***W***_***k***_ which is the best rank-*k* approximation of ***W***. Finally, NysCK decomposes $\tilde{K}$ with $\tilde{K}=FF^{T}$ where $F=[\begin{array}{c} F_{l}\\ F_{v} \end{array}]$ represents *n* linearly separable samples. Each row of ***F*** corresponds to an original sample, i.e., ***F***_***l***_ corresponds to ***X***_***l***_ while ***F***_***v***_ corresponds to ***X***_***v***_. Finally, we can train a linear classifier on ***F***_***l***_ and classify ***F***_***v***_. According to [15], computational and space complexities are *O*(*n*(*nd* + *k*^2^)) and *O*(*n*(*d* + *k*)) respectively where *d* is the dimension of each sample.

### INMKMHKS

Procedure of INMKMHKS consists of four steps. (1) For a data set ***X*** with *n* samples, INMKMHKS adopts kernel clustering to cover ***X*** with *M* clusters and samples in each cluster have same class labels. Then INMKMHKS regards midpoint and width of each cluster as parameters of a RBF kernel and as a result, *M* kernel functions are generated without setting initial kernel parameters. (2) With usage of *M* kernel functions, INMKMHKS generates *M* kernel matrices ***K***_***p***_s and gets corresponding Nyström approximation forms ${\tilde{K}}_{p}\text{s}$ without setting initial *s* (*p* = 1, 2,…, *M*). (3) INMKMHKS calculates coefficient of each ${\tilde{K}}_{p}$ and constructs ensemble kernel matrix ***G*** with ${\tilde{K}}_{p}\text{s}$ and corresponding coefficients. (4) INMKMHKS applies ***G*** into the KMHKS-based process and gets the final discriminant function.

## INysCK and MINysCK

### INysCK

#### Generating kernel functions without setting initial kernel parameters

Suppose there is a *L*-class data set ***X*** including *l* training samples ***X***_***l***_ and *v* test samples ***X***_***v***_ (*n* = *l* + *v*). Here ***X***_***l***_ = {***X***_**1**_, ***X***_**2**_,…, ***X***_***L***_} = {***x***_**1**_, ***x***_**2**_,…, ***x***_***l***_} and the class labels are ***Y*** = {*y*_1_, *y*_2_,…, *y*_*L*_}. For each class ***X***_***c***_ (*c* = 1, 2,…, *L*), it consists of *n*_*c*_ samples, i.e., $X_{c}=\left\{x_{c1},x_{c2},...,x_{cn_{c}}\right\}$. Here, *n*_*c*_ is the number of samples in ***X***_***c***_ and *l* = *n*_1_ + *n*_2_ + … + *n*_*L*_. ***x***_***cj***_ is *j*th sample of ***X***_***c***_ where *j* ∈ {1, 2, …, *n*_*c*_}. Then on the base of *l* training ones, we generate kernel functions with the following way.

For generating the first kernel function, we compute midpoint ***μ*** of all training samples, i.e., $\mu =\frac{\sum_{i=1}^{l}x_{i}}{l}$ and distance between ***x***_***i***_ and ***μ***, i.e., $d_{x_{i}}$. Distances are sorted in an ascending order, i.e., $d_{x_{\left(1\right)}}\leq d_{x_{\left(2\right)}}\leq \ldots \leq d_{x_{\left(l\right)}}$ and the corresponding samples are denoted as ***x***_(**1**)_, ***x***_(**2**)_,…,***x***_(***l***)_. If the class labels of ***x***_(**1**)_, ***x***_(**2**)_,…,***x***_(***u***)_ are same while the label of ***x***_(***u***+**1**)_ is not same as the one of ***x***_(***u***)_, then we let kernel parameters $\left(\sigma ,\mu^{{}^{\prime }}\right)=\left(d_{x_{\left(u\right)}},\mu \right)$ (in our work, used kernel function is RBF and its expression is $k\left(x_{i},\mu^{{}^{\prime }}\right)=exp\left(-\frac{∥x_{i}-\mu^{{}^{\prime }}{∥}^{2}}{2\sigma^{2}}\right)$). Then for this kernel function, we let corresponding samples ***x***_(**1**)_, ***x***_(**2**)_,…,***x***_(***u***)_ be basic samples and *u* be basic number. What’s more, in order to generate the second kernel function, we remove ***x***_(**1**)_, ***x***_(**2**)_,…,***x***_(***u***)_ from ***X***_***l***_ and repeat previous steps. We repeat the steps again and again until each training sample belongs to basic samples of one kernel function. After this generation way, we can get *M* new kernel functions, i.e., *k*_1_(***x***_***i***_, ***x***_***j***_),…, *k*_*p*_(***x***_***i***_, ***x***_***j***_),…, *k*_*M*_(***x***_***i***_, ***x***_***j***_) where *p* = 1, 2,…, *M*, we also get the corresponding *M* *σ*s and ***μ***′s.

#### Constructing kernel matrices with Nyström approximation technique

(1) We construct kernel matrices according to these *M* kernel functions. Suppose for *p*th kernel function, its parameters are $\left(\sigma_{p},\mu_{p}^{{}^{\prime }}\right)$ and basic samples are ***x***_(**1**)_,…, ***x***_(***u***)_. With all *n* samples, the corresponding *n* × *n* kernel matrix is ***K***_***p***_ = (*k*(***x***_***i***_, ***x***_***j***_))_*n*×*n*_ and its *i*th row and *j*th column element is $k\left(x_{i},x_{j}\right)=exp\left(-\frac{∥x_{i}-x_{j}{∥}^{2}}{2\sigma_{p}^{2}}\right)$ where *i*, *j* = 1,…, *n*. For convenience, in ***K***_***p***_, {***x***_**1**_,…,***x***_***l***_} corresponds to *l* training samples and {***x***_***l***+**1**_,…,***x***_***n***_} corresponds to *v* test ones.

(2) We centralize and normalize ***K***_***p***_ with Eqs (1) and (2) just for convenient calculation. Here **1**_***n***×***n***_ is a *n* × *n*-dimensional identity matrix, *trace* indicates the trace of a matrix, and we use ***K***_***p***_ to denote the centralized and normalized matrix ***K***_***trp***_ for convenience.

$$\begin{array}{c} K_{\text{cp}}=K_{p}-\frac{1}{n}1_{n\times n}K_{p}-\frac{1}{n}K_{p}1_{n\times n}+\frac{1}{n^{2}}1_{n\times n}K_{p}1_{n\times n} \end{array}$$ (1)

$$\begin{array}{c} K_{\text{trp}}=\frac{K_{\text{cp}}}{trace(K_{\text{cp}})} \end{array}$$ (2)

(3) For ***K***_***p***_, if both ***x***_***i***_ and ***x***_***j***_ are in the set {***x***_(**1**)_,…,***x***_(***u***)_}, we combine these *k*(***x***_***i***_, ***x***_***j***_)s together and generate an *u* × *u*-dimensional matrix, i.e, ***W***_***p***_. If only one of ***x***_***i***_ and ***x***_***j***_ is in this set, we combine these *k*(***x***_***i***_, ***x***_***j***_)s together and generate a (*n* − *u*) × *u*-dimensional matrix, i.e, ***K***_***p*21**_. Then $K_{p12}=K_{p21}^{T}$. If neither ***x***_***i***_ nor ***x***_***j***_ is in this set, we combine these *k*(***x***_***i***_, ***x***_***j***_)s together and generate a (*n* − *u*) × (*n* − *u*)-dimensional matrix, i.e, ***K***_***p*22**_. The relative positions of those *k*(***x***_***i***_, ***x***_***j***_)s in ***W***_***p***_, ***K***_***p*12**_, ***K***_***p*21**_, and ***K***_***p*22**_ are not changed. With above definitions, we decompose ***K***_***p***_ with Eq (3) and let *s* = *u* without initializing *s*.

$$\begin{array}{c} K_{p}=[\begin{array}{cc} W_{p} & K_{p12}\\ K_{p21} & K_{p22} \end{array}]\;and\;C_{p}=(\begin{array}{c} W_{p}\\ K_{p21} \end{array}) \end{array}$$ (3)

(4) We carry out SVD on ***W***_***p***_, i.e. $W_{p}=U_{p}\Lambda_{p}U_{p}^{T}$. Here, **Λ**_***p***_ = *diag*(*σ*_*p*1_, ⋯, *σ*_*pu*_). *σ*_*pi*_ is the *i*th largest singular value. ***U***_***p***_ is composed of the eigenvectors of the ***W***_***p***_ based on *σ*_*pi*_ and *diag* indicates the diagonalization operation.

(5) We get the rank-*k* Nyström approximation matrix for ***K***_***p***_ by

$$\begin{array}{c} {\tilde{K}}_{p}=C_{p}W_{\text{pk}}^{+}C_{p}^{T} \end{array}$$ (4)

where $W_{\text{pk}}^{+}=\sum_{i=1}^{k}\sigma_{pi}^{-1}U_{p}^{\left(i\right)}U_{p}^{(i)T}$ is the rank-*k* pseudo-inverse of ***W***_***p***_, *k* (*k* ≤ *u*) is the rank of $W_{\text{pk}}^{+}$ and $U_{p}^{\left(i\right)}$ is *i*th column of ***U***_***p***_.

(6) After repeating previous steps, we can get all ${\tilde{K}}_{p}\text{s}$ and for each ***K***_***p***_, we have a corresponding basic number *u*. Then we let largest *u* be *s*.

(7) According to [14] and Nyström approximation error between ***K***_***p***_ and ${\tilde{K}}_{p}$, we calculate coefficient *α*_*p*_ of each ${\tilde{K}}_{p}$ by Eq (5).

$$\begin{array}{c} \xi_{p}={∥{\tilde{K}}_{p}-K_{p}∥}_{F}\;and\;\alpha_{p}=\frac{e^{-\eta \xi_{p}}}{Z} \end{array}$$ (5)

where ‖.‖_*F*_ represents the Frobenius norm, *η* > 0 is a predefined parameter, $Z=\sum_{p=1}^{M}e^{-\eta \xi_{p}}$ is a normalization factor which is used to set $\sum_{p=1}^{M}\alpha_{p}=1$.

(8) Finally, we get the ensemble kernel matrix ***G*** with the following equation.

$$\begin{array}{c} G=\sum_{p=1}^{M}\alpha_{p}{\tilde{K}}_{p} \end{array}$$ (6)

#### Getting corresponding linearly separable samples

(1) Once we get ***G***, we let ***D*** = *diag*(*D*_11_,…, *D*_*nn*_) where element $D_{ii}=\sum_{j=1}^{n}G_{ij}$ and *G*_*ij*_ represents the *i*th row and *j*th column element of ***G*** (*i*, *j* = 1,…, *n*). Then we decompose ***G*** with Eq (7) where $W_{G}\in R^{s\times s}$, $G_{12}\in R^{s\times (n-s)}$, $G_{12}=G_{21}^{T}$, $G_{22}\in R^{\left(n-s)\times (n-s\right)}$, $C_{G}\in R^{n\times s}$. Since *s* is gotten with sub-step (6) in previous subsection and ***G*** is combination of multiple ${\tilde{K}}_{p}$, so elements in ***W***_***G***_, ***G***_**12**_, ***G***_**21**_, ***G***_**22**_ are fixed here. Since *s* < *l*, *n* − *s* = *l* + *v* − *s* > *v*, so *v* × *v* part in the lower right corner of ***G***_**22**_ corresponds to *v* test samples.

$$\begin{array}{c} G=[\begin{array}{cc} W_{G} & G_{12}\\ G_{21} & G_{22} \end{array}]\;and\;C_{G}=(\begin{array}{c} W_{G}\\ G_{21} \end{array}) \end{array}$$ (7)

(2) We carry out SVD on ***W***_***G***_, i.e. $W_{\text{Gk}}=U_{W_{G},k}\Sigma_{W_{G},k}U_{W_{G},k}^{T}$ where ***W***_***Gk***_ is the best rank-*k* approximation of ***W***_***G***_, **Σ**_***W***_***G***_,***k***_ is a diagonal matrix and diagonal consists of first *k* approximate eigenvalues, and ***U***_***W***_***G***_,***k***_ consists of the corresponding *k* approximate eigenvectors. Then we get Nyström approximation matrix $\tilde{G}$ of ***G*** with Eq (8) where $W_{Gk}^{†}$ denotes the pseudo-inverse of ***W***_***Gk***_.

$$\begin{array}{c} C_{G}W_{Gk}^{†}C_{G}^{T}\approx \tilde{G} \end{array}$$ (8)

(3) After that, we compute **Σ**_***k***_ and ***U***_***k***_ using Eq (9) where $\Sigma_{W_{G},k}^{†}$ is the pseudo-inverse of **Σ**_***W***_***G***_,***k***_. In terms of each element *σ*_*i*_ of **Σ**_***k***_, we apply Eq (10) and get the $\tilde{\Sigma_{k}}=diag\left(\varphi \left(\sigma_{1}\right),\ldots ,\varphi \left(\sigma_{k}\right)\right)$. Generally speaking, since *v* is larger than 9, so we use *h* = *l* + 9 is feasible.

$$\begin{array}{c} \Sigma_{k}=\left(\frac{n}{s}\right)\Sigma_{W_{G},k}\;and\;U_{k}=\sqrt{\frac{s}{n}}C_{G}U_{W_{G},k}\Sigma_{W_{G},k}^{†} \end{array}$$ (9)

$$\begin{array}{c} \varphi \left(\sigma_{i}\right)=\{\begin{array}{ccc} \sqrt{\sigma_{i}} & & i<h(=l+9)\\ \sigma_{i}^{2} & & i\geq h(=l+9) \end{array} \end{array}$$ (10)

(4) Once we get $\tilde{\Sigma_{k}}$, we let $\tilde{L}\approx D^{-1/2}U_{k}\tilde{\Sigma_{k}}U_{k}^{T}D^{-1/2}$ and then ${\tilde{L}}^{1/2}\approx D^{-1/2}U_{k}{\left(\tilde{\Sigma_{k}}\right)}^{1/2}$. Then, we have ${\tilde{D}}_{ii}=1/{\tilde{L}}_{ii}$ and get $\tilde{D}=diag\left({\tilde{D}}_{11},\ldots ,{\tilde{D}}_{nn}\right)$ where ${\tilde{L}}_{ii}$ is *i*th row and *i*th column element of $\tilde{L}$. Finally, we can get ${\tilde{D}}^{1/2}$ and linearly separable data set $F=[\begin{array}{c} F_{l}\\ F_{v} \end{array}]$ by Eq (11). According to [15] said, each row of ***F*** corresponds to an original sample, i.e., ***F***_***l***_ corresponds to ***X***_***l***_ and ***F***_***v***_ corresponds to ***X***_***v***_. Once ***F*** is gotten, we can adopt linear classifiers to train and classify them. For convenience, Table 2 shows framework of INysCK.

$$\begin{array}{c} F\approx {\tilde{D}}^{1/2}{\tilde{L}}^{1/2} \end{array}$$ (11)

**Table 2:** Algorithm: INysCK.

**Input**: *L*-class data set ***X*** = **[*X***_***l***_, ***X***_***v***_**]**. ***X***_***l***_ consists of *l* training samples and ***X***_***v***_ consists of *v* test samples. Here, *n* = *l* + *v*
1. Generate *M* kernel functions.
2. For p = 1,2,…,M do
3. Construct kernel matrix ***K***_***p***_ with *p*th kernel function.
4. Centralize, normalize, and decompose ***K***_***p***_ with Eqs (1) and (3).
5. Carry out SVD on ***W***_***p***_ and get the rank-*k* Nyström approximation matrix ${\tilde{K}}_{p}$ for *K*_*p*_ with Eq (4).
6. End for
7. Compute coefficient *α*_*p*_ of each ${\tilde{K}}_{p}$ with Eq (5) and get ensemble kernel matrix ***G*** with Eq (6).
8. On the base of ***G***, get ***D*** and decompose ***G*** with Eq (7).
9. Carry out SVD on ***W***_***G***_ and get Nyström approximation matrix $\tilde{G}$ of ***G*** with Eq (8).
10. Compute ***Σ***_***k***_, ***U***_***k***_ and get $\tilde{\Sigma_{k}}$ with Eqs (9) and (10).
11. Get ${\tilde{L}}^{1/2}$ and ${\tilde{D}}^{1/2}$ and obtain ***F*** with Eq (11).
**Output**: $F=[\begin{array}{c} F_{l}\\ F_{v} \end{array}]$ and ***F***_***l***_ corresponds to ***X***_***l***_ while ***F***_***v***_ corresponds to ***X***_***v***_

### MINysCK

Suppose there is a multi-view data set $X={\left\{X^{g}\right\}}_{g=1}^{V}={\left\{x_{i}\right\}}_{i=1}^{n}$ where *V* is the number of views and *n* is the number of samples. The *g*th view is $X^{g}={\left\{x_{i}^{g}\right\}}_{i=1}^{n}$ and the *i*th sample is $x_{i}={\left\{x_{i}^{g}\right\}}_{g=1}^{V}$. $x_{i}^{g}$ represents *g*th view of *i*th sample. For each view ***X***^***g***^, its dimension is *d*^*g*^ which indicates that this view consists of *d*^*g*^ features. Now in the procedure of MINysCK, we conduct INysCK on each view ***X***^***g***^ and get the corresponding ***F***, i.e., ***F***^***g***^. Then for *V* views, we get *V* groups ***F***. For the ***X***, its linear form is ***F*** = **{*F***^**1**^,***F***^**2**^,…,***F***^***V***^**}**. Finally, we can adopt some multi-view classifiers to process ***F***. Table 3 shows framework of MINysCK.

**Table 3:** Algorithm: MINysCK.

**Input**: multi-view data set $X={\left\{X^{g}\right\}}_{g=1}^{V}={\left\{x_{i}\right\}}_{i=1}^{n}$
1. For g = 1,2,…,V do
2. Change ***X***^***g***^ to corresponding ***F***^***g***^ with INysCK.
3. End for
4. Obtain ***F*** = **{*F***^**1**^,***F***^**2**^,…,***F***^***V***^**}**.
**Output**: ***F***

### Computational complexity and space complexity

According to [15], the computational complexity and space complexity of NysCk are *O*(*n*(*nd* + *k*^2^)) and *O*(*n*(*d* + *k*)) respectively where *d* is the dimension of each sample. Then compared with NysCk, the main added steps of INysCK are the generation of kernel functions and matrices. Thus, the added computational complexity is *O*(*Ml*^2^) and the added space complexity is *O*(*Ml*^2^). Since in real-world applications, *M* ≪ *n* and *l* ≪ *n*, so computational complexity and space complexity of INysCk are almost same as ones of NysCK. For MINysCK, the computational complexity is $\sum_{g=1}^{V}O\left(n\left(nd^{g}+k^{2}\right))=O(n(nd+k^{2})\right)$, the space complexity is $\sum_{g=1}^{V}O\left(n\left(d^{g}+k\right))=O(n(d+k)\right)$. Thus, we find that computational and space complexities of INysCK and MINysCK are same as the ones of NysCK.

## Experiments

### Experimental setting

We adopt four multi-view data sets (NUS-WIDE, YMVG, DBLP, Cora) and four UCI machine learning repository (UCI) [23] data sets (YCS, AA, BC, Arrhythmia) for experiments in niche targeting. Among these data sets, half of them are large-scale and the left are small-scale. Information of used UCI data sets is given in Table 4 and in terms of four multi-view ones, we describe them as below where *D* denotes dimensionality. (1) NUS-WIDE is a web image data set which consists of 269648 samples (images), 5018 classes, and 6 views [24]. The six views are color histogram (Col-h, 64-D), color correlogram (Col-c, 144-D), edge direction histogram (Ed-h, 73-D), wavelet texture (Wav, 128-D), block-wise color moments (Bw-cm, 225-D), and bag of words based on SIFT descriptions (B-SIFT, 500-D); (2) YMVG [25] is the abbreviation of YouTube multi-view video games and it consists of 120000 samples (videos) from 31 classes (games). Each sample consists of 13 views. They are audio mfcc (A-m, 2000-D), audio sai boxes (A-s-b, 7168-D), audio sai intervalgrams (A-s-i, 4096-D), audio spectrogram stream (A-s-s, 1024-D), audio volume stream (A-v-s, 64-D), text description unigrams (T-d-u, 558936-D), text game lda 1000 (T-g-l, 1000-D), text tag unigrams (T-t-u, 422627-D), vision cuboids histogram (V-c-h, 512-D), vision hist motion estimate (V-h-m-e, 64-D), vision hog features (V-h-f, 647-D), vision hs hist stream (V-h-h-s, 1024-D), and vision misc (V-m, 838-D); (3) DBLP [26, 27] is abbreviation of digital bibliography and library project. Original DBLP is very large and we select 5000 samples from 4 classes for experiments. Each sample has two views, one is paper name (P-n, 6167-D) and the other is term (Te, 3787-D); (4) Cora [27, 28] is adapted from original Cora data set [29] and it consists of 12004 scientific articles (samples) from 10 thematic classes and for each sample, it has two views, i.e., content (Co, 292-D) and relational (Re, 12004-D). What’s more, these data sets are third party ones and others would be able to access these data in the same manner as what we have done. Moreover, we confirm that we don’t have any special access privileges that others would not have.

**Table 4:** Description of the used UCI data sets.

Data set	No. dimensions	No. classes	No. samples
YouTube Comedy Slam (YCS)	2	2	1138562
Authorship Attribution (AA)	1000	50	93600
Breast Cancer (BC)	10	2	699
Arrhythmia	279	16	452

Then we adopt CK, NysCK, INysCK, and MINysCK to change nonlinearly separable samples to linearly ones. If we use original data sets for experiments, we adopt ‘Null’ for representation. Here, we treat CK as a baseline method and if we only compare with NysCK, NysCK can be regarded as a baseline one.

We use classifiers shown in Table 5 for further processing and linear classifiers are only feasible for linearly separable data sets while nonlinear classifiers are feasible for both nonlinearly and linearly ones. Similarly, multi-view classifiers can process not only multi-view but also single-view data sets while single-view classifiers are only feasible for single-view ones. We adopt SVM and MSVM as two baseline classifiers in respective experiments.

**Table 5:** Used classifiers.

	nonlinear	linear
single-view	KMHKS [4], KSVM [5]	SVM [1], MHKS [1]
	NDL [34], LCDL [35]	BPDMatMHKS [36], rWLSSVC [37]
multi-view	MultiV-KMHKS [6], DLMMLM [9]	MSVM [38], MLRSSC [39]
	MGGM [27], MV-LSSVM [40]	
	MVMFL [41], SMVMED [42]	
	KMLRSSC [39], MVKSC [43]	

What’s more, for each data set, 70% of samples are chosen in random as training samples and the remaining are for test. In order to get the truly experimental results, we adopt 10-fold cross validation strategy [10]. Moreover, one-against-one classification strategy is used for multi-class problems here [30–33]. In order to get the average experimental results, we repeat the experiments for 10 times. The computations are performed on Intel Core 4 processors with 2.66GHz, 4G RAM DDR3, Win 7, and MATLAB 2014 environment.

### Independent experiments

This part shows the performances of our proposed methods on different kinds of data sets.

#### Accuracy comparison on large-scale single-view data sets

**Fig 1:**
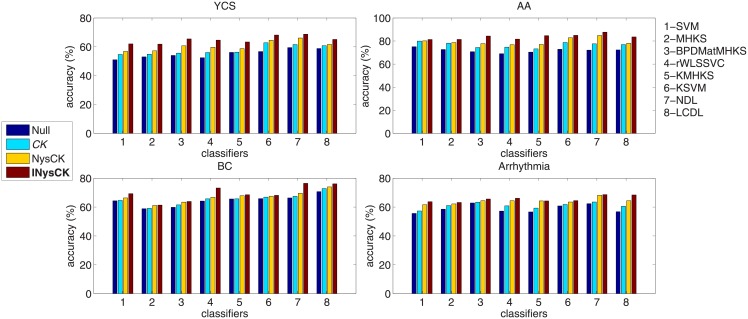
Accuracy with related classifiers and CK-based methods on used single-view data sets. CK-related method in italic represents baseline one and one in bold denotes the proposed one. For classifiers, SVM is used as the baseline one and we just clarify this point in words rather than in font. In other figures and tables, we have similar representations.

First, we show effectiveness of our INysCK on two large-scale single-view data sets YCS and AA. For fair comparison, we select 8 single-view classifiers shown in Table 5 for experiments and adopt CK, NysCK, INysCK to change samples into linearly separable ones. Moreover, as we know, accuracy, true positive rate (*acc*^+^), true negative rate (*acc*^−^), positive predictive value (PPV), F-Measure, G-Mean, etc. [44] are widely used to evaluate the classification performances. Here, we only show the results about accuracy due to for other evaluation criteria, we draw similar conclusions. Then the top two sub-figures of Fig 1 show the results. According to these two sub-figures, we can see that for large-scale single-view data sets, our INysCK brings a best accuracy no matter which classifier is used. Specially, we find compared with CK and NysCK, for the case AA with SVM, the improvement of INysCK is little while for other cases, the improvement is more. In order to elaborate this phenomenon, we analysis the distributions of YCS and AA. Since these two data sets are large-scale, so we won’t show the distributions of their samples with figure and just describe the distributions in short. We find that for these two data sets, their samples distribute with an interlaced way and a high nonlinearity. After carrying out CK-related methods, most samples become linearly separable and compared with CK and NysCK, with INysCK used, samples have a higher linearity. Moreover, since the sizes of YCS and AA are large, so the advantages of linearities derived from INysCK are larger. Thus when we use those single-view classifiers no matter nonlinear ones and linear ones to process the changed samples, accuracies are higher. In terms of the case AA with SVM, we find with CK, NysCK, INysCK used, the classification functions provided by support vectors are similar. That’s why that our INysCK brings a little improvement for this case.

#### Accuracy comparison on small-scale single-view data sets

Second, we adopt data sets BC and Arrhythmia for experiments so as to validate effectiveness of INysCK on small-scale single-view problems. Similarly with the above experiments, single-view classifiers shown in Table 5, and CK, NysCK are adopted. Then bottom two sub-figures of Fig 1 show the results. According to these two sub-figures, it is found that for small-scale single-view problems, our INysCK still brings a best accuracy in average. But compared with the results given in above experiments, on more cases, INysCK only brings a little improvement and we find for the case Arrhythmia with KMHKS, NysCK outperforms INysCK. For this phenomenon, we also analysis the distributions of BC and Arrhythmia. We find that with INysCK used, samples of these two data sets are linearly separable. But since the sizes of them are small, so advantages of linearities derived from INysCK are not obvious even more not exist. Thus for some cases, the improvement of INysCK is little and for the case Arrhythmia with KMHKS, INysCK performs worse than NysCK due to the advantage of linearity derived from INysCK is not exist in terms of KMHKS.

#### Accuracy comparison on large-scale multi-view data sets

**Fig 2:**
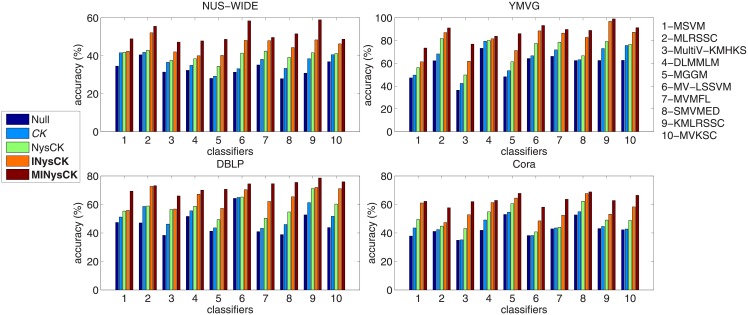
Accuracy with related classifiers and CK-based methods on used multi-view data sets. CK-related method in italic represents baseline one and ones in bold denote the proposed ones. For classifiers, MSVM is used as the baseline one and we just clarify this point in words rather than in font. In other figures and tables, we have similar representations.

Then, we use NUS-WIDE and YMVG to show the effectiveness of proposed methods on large-scale multi-view problems. The used classifiers are multi-view ones shown in Table 5. Then here, we use CK, NysCK, INysCK, and MINysCK to change samples. Although NUS-WIDE and YMVG are multi-view data sets, in order to carry out CK, NysCK, and INysCK, we regard all views as a whole view. The top two sub-figures of Fig 2 show the results. According to these two sub-figures, we find that as a multi-view method, our MINysCK brings a best accuracy in average and the improvement is much more than the previous results, especially, for the cases NUS-WIDE with MSVM (MultiV-KMHKS, DLMMLM, MGGM, MV-LSSVM, SMVMED, KMLRSSC) and YMVG with MSVM (MultiV-KMHKS, MGGM). The main reason is that for multi-view data sets, MINysCK is more feasible than the single ones including CK, NysCK, and INysCK. What’s more, taking all views as a whole view to carry out CK, NysCK, and INysCK cannot reflect the differences among views and this operation can only keep the linearity of samples on the whole view and cannot promise linearity on respective view. That’s why MINysCK performs best on NUS-WIDE and YMVG.

#### Accuracy comparison on small-scale multi-view data sets

Now we adopt DBLP and Cora for the accuracy comparison on small-scale multi-view problems and other settings are same as ones given in above experiments. The bottom two sub-figures of Fig 2 show the results. From these two sub-figures, we can see that our MINysCK performs best on small-scale multi-view data sets as well. But compared with the above experimental results in careful, we find since the sizes of DBLP and Cora are more smaller than the ones of NUS-WIDE and YMVG, so advantages of linearities derived from MINysCK are not obvious, as a result, the improvement of MINysCK has a reduction.

#### Comparison about time cost

Besides the accuracy comparisons, we show the time cost comparison here. As we said before, the computational complexity and space complexity of INysCK and MINysCK are same as the ones of NysCK, i.e., *O*(*n*(*nd* + *k*^2^)) and *O*(*n*(*d* + *k*)) respectively where *d* is the dimension of each sample and all of these three NysCK-related methods are used to change the nonlinearly separable samples to the linearly separable ones. Here, we show the practice time of them on different data sets in Table 6 and from this table, it is found that (1) since the procedures of INysCK and MINysCK are more complicate than NysCK, so they both cost longer time in average while the increased time is acceptable; (2) for multi-view data sets, MINysCK costs less time than INysCK, we think the main reason is that we won’t regard the multiple views as a single whole view with some fusion techniques and process each view in each small problem. This maybe brings a smaller total time cost.

**Table 6:** Comparison about time (in seconds) cost for the three NysCK-related methods.

Data set	*NysCK*	INysCK	MINysCK	Data set	*NysCK*	INysCK	MINysCK
YCS	43.405	44.642	/	AA	146.672	154.527	/
BC	0.087	0.095	/	Arrhythmia	1.577	1.634	/
NUS-WIDE	1380.392	1434.962	1406.573	YMVG	241078.011	249127.624	247240.890
DBLP	4.166	4.282	4.266	Cora	29.663	32.586	32.069

### Comprehensive experiments

This part shows average performances of our proposed methods on all used data sets.

#### Distributions of samples with different CK-related methods

**Fig 3:**
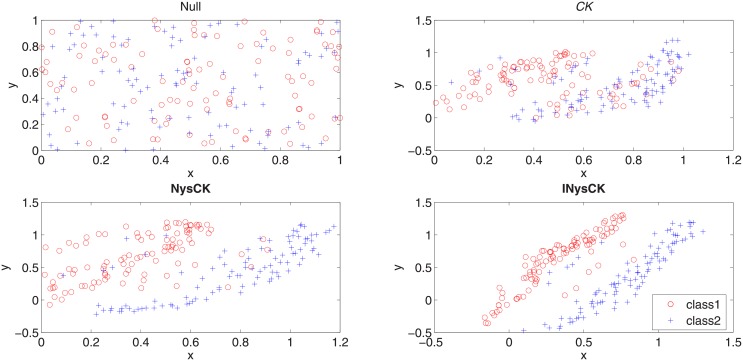
Distributions of samples with different CK-related methods on a binary-class data set.

Here, we use a two-dimensional binary-class data set ***X*** to compare the performances of CK, NysCK, and INysCK when they change nonlinearly separable data to linearly ones. The distributions of samples before or after carrying out CK-related methods are shown in Fig 3. According to this figure, it is found that (1) all CK-related methods can change nonlinearly separable samples to linearly separable ones at some extends; (2) with INysCK used, for the same class, most samples locate in an area centrally and only few samples locate far from this area. With calculation, we find that with CK used, 20% samples locate in the area which belongs to different classes. For NysCK, the ratio is 6.5% while for INysCK, the ratio is only 3.5%. This means with our methods used, samples have a higher linearity. What’s more, since it is always hard for people to show a multi-view data set or multi-dimensional data set whose dimensionality is large than two with a two-dimensional picture, thus we won’t show the distribution of samples with MINysCK used and only use a two-dimensional data set for experiments here. But this won’t influence our conclusion.

#### Convergence analysis

Convergence is an important criterion to assess the effectiveness of a classifier and if a classifier can converge within limited iterations with a better classification performance, we say this classifier is effective. What’s more, the distribution of samples also affect the convergence and samples with a high linearity always accelerate the optimization of a classifier. Here, we adopt an empirical justification given in [45] to measure the convergence of classifiers with our methods used and Table 7 shows the results. Each cell in this table denotes the average number of iterations of a classifier on all used data sets with a CK-related method used. According to this table and combining the results given before, we know that with our proposed INysCK and MINysCK used, the changed samples have a higher linearity and these samples accelerate the optimization of classifiers which indicates a smaller numbers of iterations. What’s more, since MINysCK is more feasible for multi-view data sets, so for the multi-view classifiers, they can converge faster with MINysCK used.

**Table 7:** The numbers of iterations comparisons.

single-view	Null	*CK*	NysCK	INysCK	multi-view	Null	*CK*	NysCK	INysCK	MINysCK
SVM	17.20	17.13	16.06	14.96	MSVM	17.57	16.35	14.72	14.44	13.43
MHKS	21.30	19.69	18.79	18.06	MLRSSC	29.18	26.60	24.44	23.26	22.05
BPDMatMHKS	22.94	22.94	22.29	21.89	MultiV-KMHKS	25.63	24.77	22.71	21.24	20.16
rWLSSVC	20.13	19.30	18.96	18.64	DLMMLM	28.91	27.10	25.68	24.84	23.18
KMHKS	20.82	18.98	17.55	17.13	MGGM	27.03	24.46	23.62	22.07	21.26
KSVM	17.03	16.60	15.53	15.29	MV-LSSVM	43.56	40.94	38.92	37.87	36.39
NDL	19.82	18.92	18.34	17.46	MVMFL	66.96	66.33	64.75	62.91	62.31
LCDL	22.42	21.81	19.92	19.26	SMVMED	37.19	33.95	31.37	29.06	27.69
					KMLRSSC	32.69	32.65	32.00	30.33	28.16
					MVKSC	26.78	25.55	23.48	21.46	20.70

#### Rademacher complexity analysis

**Fig 4:**
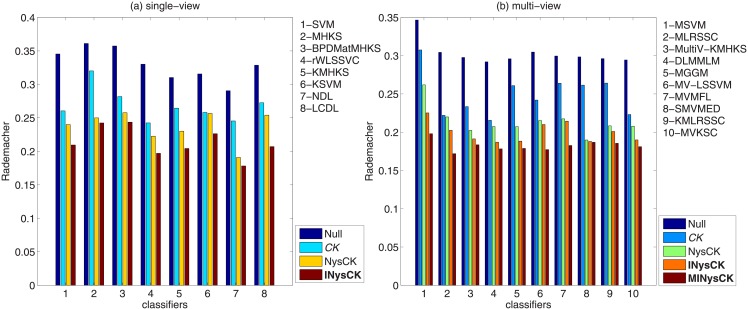
The average Rademacher complexity comparison.

As [14] and [11] said, Rademacher complexity is a reflection about generalization risk bound and performance behavior of a classifier. A smaller Rademacher complexity indicates a better performance of a classifier and a lower generalization risk bound. Here, we adopt the same method given in [11] to compute Rademacher complexity for classifiers with different CK-related methods used. Fig 4 shows the results and according to this figure, we know (1) in terms of single-view classifiers, since samples with INysCK used have a higher linearity, so classifiers have smaller Rademacher complexities; (2) in terms of multi-view classifiers, since MINysCK is more feasible and it also makes the samples have a higher linearity, thus related Rademacher complexities are smaller.

#### Significance analysis

We adopt Friedman-Nemenyi statistical test [46] to validate the difference between our proposed methods and the previous work is significant. In terms of Friedman-Nemenyi statistical test, Friedman test is used to analyze if the differences between all compared algorithms on multiple data sets are significant or not while Nemenyi test is used to analyze if the differences between two compared algorithms on multiple data sets are significant or not.

In order to carry out Friedman test, we treat each CK-related method as an ‘algorithm’ and regard each classifier as a ‘data set’. Then according to the average accuracy of an ‘algorithm’ on a ‘data set’, Friedman test ranks the ‘algorithm’s for each ‘data set’ as shown in Table 8. (1) For single-view cases, since we use 4 ‘algorithm’s and 8 ‘data set’s, we carry out Friedman test and get $\chi_{F}^{2}=21.47$ and *F*_*F*_ = 59.37 (the computation equations of $\chi_{F}^{2}$ and *F*_*F*_ can be found in [46]). Further, with 4 ‘algorithm’s and 8 ‘data set’s, *F*_*F*_ is distributed according to the *F* distribution with 4 − 1 = 3 and (4 − 1) × (8 − 1) = 21 degrees of freedom. The critical value of *F*_0.05_(3, 21) when *α* = 0.05 is 3.0725 and *F*_0.10_(3, 21) when *α* = 0.10 is 2.3649. As *F*_*F*_ > 3.0725 and *F*_*F*_ > 2.3649, we say for the single-view cases, the differences between all compared CK-related methods on multiple classifiers are significant. (2) Similarly, for multi-view cases, with 5 ‘algorithm’s and 10 ‘data set’s used, related $\chi_{F}^{2}=35.06$, *F*_*F*_ = 63.91, *F*_0.05_(4, 36) = 2.6335, and *F*_0.10_(4, 36) = 2.1079. Since *F*_*F*_ > 2.6335 and *F*_*F*_ > 2.1079, we can draw a conclusion that for the multi-view cases, the differences between all compared CK-related methods on multiple classifiers are also significant.

**Table 8:** Average rank comparisons for different CK-related methods and classifiers.

single-view	Null	*CK*	NysCK	INysCK	multi-view	Null	*CK*	NysCK	INysCK	MINysCK
SVM	3.25	3.75	1.75	1.25	MSVM	4.80	4.00	3.20	1.60	1.40
MHKS	3.00	4.00	2.00	1.00	MLRSSC	5.00	3.60	3.40	1.40	1.60
BPDMatMHKS	3.25	3.75	1.75	1.25	MultiV-KMHKS	5.00	3.60	3.40	1.60	1.40
rWLSSVC	3.25	3.75	1.75	1.25	DLMMLM	5.00	3.60	3.40	1.60	1.40
KMHKS	3.00	4.00	2.00	1.00	MGGM	4.80	4.00	3.20	1.60	1.40
KSVM	3.25	3.75	1.75	1.25	MV-LSSVM	5.00	3.60	3.40	1.60	1.40
NDL	3.25	3.75	1.75	1.25	MVMFL	4.60	4.20	3.20	1.60	1.40
LCDL	3.00	4.00	2.000	1.000	SMVMED	5.00	3.60	3.40	1.60	1.40
Average	3.16	3.84	1.84	1.16	KMLRSSC	4.80	3.80	3.40	1.60	1.40
					MVKSC	4.80	3.80	3.40	1.40	1.60
					Average	4.88	3.78	3.34	1.56	1.44

Then we use Nemenyi test for pairwise comparisons. (1) For single-view cases, when *α* = 0.05, the critical value *q*_0.05_ is 2.569 (see Table 9) and the corresponding *CD* is $2.569\sqrt{\frac{4\cdot (4+1)}{6\cdot 8}}=1.66$. When *α* = 0.10, the critical value *q*_0.10_ is 2.291 (see Table 9) and the corresponding *CD* is $2.291\sqrt{\frac{4\cdot (4+1)}{6\cdot 8}}=1.48$. Then according to the principle of Nemenyi test, since 1.84 < 1.16 + 1.66 = 2.82 < 3.84 and 1.84 < 1.16 + 1.48 = 2.64 < 3.84, so we say the differences between INysCK and CK (NysCK) are (not) significant. (2) For multi-view cases, according to Table 9, since *q*_0.05_ = 2.728 and *q*_0.10_ = 2.459, the corresponding *CD*s are $2.728\sqrt{\frac{5\cdot (5+1)}{6\cdot 10}}=1.93$ and $2.459\sqrt{\frac{5\cdot (5+1)}{6\cdot 10}}=1.74$ respectively. Then since 1.56 < 3.34 < 1.44 + 1.93 = 3.37 and 1.56 < 1.44 + 1.74 = 3.18 < 3.34, we say the differences between MINysCK and CK are significant and the ones between MINysCK and NysCK are significant to a certain extent.

**Table 9:** Critical values for the two-tailed Nemenyi test.

No. algorithms	2	3	4	5	6	7	8	9	10
*q*_0.05_	1.960	2.343	2.569	2.728	2.850	2.949	3.031	3.102	3.164
*q*_0.10_	1.645	2.052	2.291	2.459	2.589	2.693	2.780	2.855	2.920

As a summary, we can draw a conclusion that according to Friedman-Nemenyi statistical test, our proposed INysCK (or MINysCK) is an improvement on previous work CK (or NysCK) statistically.

#### Influence of ratio of training samples

**Fig 5:**
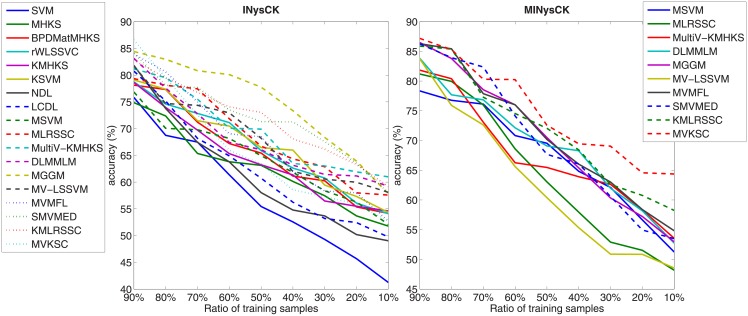
Average influence of ratio of training samples on accuracy with INysCK and MINysCK and corresponding classifiers used.

In the previous experiments, for each data set, we randomly choose 70% of samples as training part and the remaining as test part. Here, we change the ratio of training samples and show its average influence on accuracy with Fig 5. According to this figure, it is found that with the increasing of the ratio of training samples, the average accuracy also boosts.

## Conclusions and future work

### Conclusions

Traditional nonlinear classifiers are developed to process nonlinearly separable data sets and they always use kernel functions to generate several kernel matrices. After the optimization of these matrices, the optimal classifier parameters can be gotten. While one always costs high computational and space complexities to compute and store these matrices, so in order to reduce the complexities, people develop INMKMHKS which adopts Nyström approximation technique and NysCK which changes nonlinearly separable samples to linearly ones. In this work, we combine ideas of them in together to develop INysCK and MINysCK to reduce the complexities further and process single-view data sets and multi-view data sets respectively. In order to validate the effectiveness of them, we use CK and NysCK for comparisons. Then we adopt some large-scale, small-scale, single-view, multi-view data sets and single-view, multi-view, nonlinear, linear classifiers for experiments in niche targeting. Corresponding experiments about accuracy, time cost, convergence, Rademacher complexity, and so on have validated the effectiveness of INysCK and MINysCK.

According to experimental results, we can draw the following conclusions. (1) INysCK and MINysCK can change nonlinearly separable samples to be linearly separable with higher linearities and the accuracies of corresponding classifiers boost. (2) Compared with NysCK, INysCK and MINysCK both cost longer time in average while the increased time is acceptable. (3) With INysCK and MINysCK used, classifiers can converge faster and their Rademacher complexities are smaller. (4) INysCK (or MINysCK) is an improvement on previous work CK (or NysCK) statistically.

### Future work

Although our proposed methods perform better for nonlinear classification problems, according to [47] said, Nyström approximation technique is data-dependent even though we adopt the Nyström approximation technique used in INMKMHKS to avoid parameter setting problems. In [47], on the base of Hellinger’s kernel and *χ*^2^ kernel, scholars use two mapping functions which are both data-independent to enhance the classification performance. Thus, in our future work, we try to introduce the idea of [47] to our work. In other words, we will try to use data-independent mapping functions to change the nonlinearly separable samples to the linearly separable ones. What’s more, besides what we have discussed in this work, there are some other pattern recognition fields attract scholars to research, for example, unsupervised feature selection [48, 49] and multi-label learning [50]. So in our future work, we will try to introduce our methods into these fields.
